# Climate, Not Soil, Drives the Distribution of Two Closely Related Worm Lizards

**DOI:** 10.1002/ece3.72008

**Published:** 2025-08-15

**Authors:** Henrique J. Oliveira, Karoline Ceron, Mario R. Moura, Henrique C. Costa

**Affiliations:** ^1^ Programa de Pós‐Graduação em Biodiversidade e Conservação da Natureza Universidade Federal de Juiz de Fora Juiz de Fora Brazil; ^2^ Laboratório de Interações Ecológicas e Biodiversidade (LIEB), Departamento de Biologia Universidade Federal do Ceará Fortaleza Brazil; ^3^ Departamento de Sistemática e Ecologia Universidade Federal da Paraíba João Pessoa Brazil; ^4^ Departamento de Zoologia Universidade Federal de Juiz de Fora Juiz de Fora Brazil

**Keywords:** *Amphisbaena*, biogeography, coexistence, fossoriality, niche modeling, reptiles

## Abstract

A central challenge in ecology is understanding how closely related species coexist, and sister species—with which they share a closely related evolutionary history—provide a powerful system for testing niche overlap and segregation. For fossorial organisms, the relative role of climate and soil in shaping distributions remains unclear, despite their potential to drive habitat suitability and species interactions underground. Here, we examine factors driving coexistence of two sister species of worm lizards, *Amphisbaena bolivica* and *A. camura* , which show distributions mostly parapatric but with partial overlap (sympatry). We used Maxent niche models to assess how climatic, edaphic, and relief variables influence their occurrences, and also evaluated their niche overlap in the environmental spaces defined by climate and soil. The projected habitat suitability for *A. bolivica* and *A. camura* closely aligns with their known occurrence records in Argentina, Bolivia, Brazil, and Paraguay. Climatic variables were more important than edaphic and relief variables for the distribution of both species. Isothermality was the most important variable (67% percentage contribution to the Maxent ecological niche model) for *A. bolivica* , followed by mean temperature of the wettest quarter (43%). For *A. camura* , mean diurnal range was the most important (64%) followed by mean temperature of the wettest quarter (27%). We found low niche overlap in the climate space and higher than expected similarity in the edaphic space. Our findings suggest conservatism of the edaphic space occupied by these sister species, with climatic factors underlying spatial segregation among fossorial organisms.

## Graphical abstract

In this paper, we used ecological niche models to explore the coexistence of two *Amphisbaena* sister species and assess how climatic, edaphic, and relief variables influence their distributions. Additionally, we evaluated the niche overlap between the two species. Our findings demonstrate that phylogenetic relationships do not necessarily reflect niche similarity, as closely related species may adapt to different microhabitats.

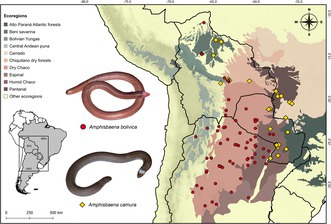

## 1 Introduction

Understanding species biogeography begins with defining and delineating basic distribution patterns (Carvalho 2010; Rodríguez et al. 2018), yet knowledge gaps persist due to limited data on species biology and geography (Hortal et al. 2015). These gaps are exacerbated by biases in research effort, which influence how ecological and geographic space are cataloged across organisms (Mazerolle et al. 2007; Kéry and Schmidt 2008; Moura et al. 2024). Such knowledge shortfalls are particularly pronounced among fossorial and semi‐fossorial species (Guedes et al. 2018; Guedes et al. 2023), including worm lizards (amphisbaenians), limbless reptiles whose cryptic burrowing lifestyle has hindered data collection on their ecology and evolution (Böhm et al. 2016; Colli et al. 2016), compromising our understanding of their biogeography.

One way to mitigate knowledge gaps on worm lizards biogeography is through using Ecological Niche Models (ENMs). These models relate occurrence data with environmental predictors (e.g., climatic and edaphic variables) to quantify suitable areas and occupied niche space (Warren et al. 2008; Franklin 2010). Ultimately, ENMs provide a framework for identifying factors that determine the spatial distribution of organisms and promote species coexistence (Schoener 1974; Chase and Leibold 2003; Fuentes‐Montejo et al. 2024). Due to niche conservatism—the tendency of closely related species to maintain similar ecological niches over time (Peterson et al. 1999; Alexandre et al. 2017)—sister species (those sharing a recent common ancestor) are particularly useful for testing hypotheses on niche overlap, segregation, and interspecific competition, as their shared evolutionary history shapes similar physiological tolerances and, consequently, their coexistence patterns (Luiselli 2006; Losos 2008; Duré et al. 2009; Ritter et al. 2021).

In worm lizards, fossoriality can directly influence species coexistence by limiting opportunities for ecological segregation (Civantos et al. 2003; Li and Wiens 2022; Anelli et al. 2024). Subterranean habitats exhibit limited vertical stratification, increasing the chances of interspecific competition and predation pressure among fossorial species compared to arboreal or terrestrial organisms (Kubiak et al. 2015; Oliveira and Scheffers 2019; Moura et al. 2023). However, burrowing behavior also shapes a distinct suite of unique biological adaptations that facilitate the fossorial lifestyle, which may conversely constrain niche segregation (Vidal et al. 2008). Indeed, fossoriality has been associated with niche conservatism and slower diversification rates in reptiles and amphibians (Bars‐Closel et al. 2017; Cyriac and Kodandaramaiah 2018; Moen and Wiens 2017). Consequently, fossorial organisms may show greater niche overlap in dimensions strongly tied to underground microhabitats, such as edaphic conditions, but weaker constraints on climatic factors, potentially facilitating niche divergence.

To explore how fossoriality, niche conservatism, and vertical partitioning shape species coexistence, we examine *Amphisbaena bolivica* Mertens, 1929, and *Amphisbaena camura* Cope, 1862, two medium‐sized sister species with parapatric distribution and partial sympatry. Although these species exhibit slight morphological differences (Gans 1965; Montero 1996a) and genetic sampling remains limited, current evidence supports their status as valid sister taxa (Teixeira‐Junior et al. 2019; Graboski et al. 2022). Their parapatry and overlapping environmental niches raise questions about the mechanisms allowing their coexistence. For instance, high niche overlap could suggest that *A. bolivica* and *A. camura* represent a single taxonomic entity. Here, we assess the environmental drivers of their geographic distributions and the factors facilitating their coexistence in shared habitats.

## 2 Methods

### 2.1 Data Source

We compiled occurrence data for *A. bolivica* and *A. camura* from literature (e.g., Montero 1996b; Cacciali et al. 2016); we searched for the species names in Google Scholar. We also reviewed all issues of the journal Herpetological Review and our personal libraries (Appendix S1). For records lacking precise coordinates, we georeferenced localities using gazetteers (e.g., Cacciali et al. 2016; Paynter 1989, 1992, 1995) and Google Earth version 7.3 (Lisle 2006). We supplemented these data with expert‐verified iNaturalist records, confirmed by one of us (H.C.C.). Although *A. bolivica* and *A. camura* are morphologically very similar, they can be distinguished by the total number of body and tail rings (more than 225 in *A. bolivica* and fewer than 200 in *A. camura* ) (Montero 1996a). If the head is completely white, the specimen can be confidently identified as *A. bolivica* (Montero 1996a). In many photographs available on iNaturalist, at least one of these diagnostic features can be observed to confirm species identification. When these characters are not visible and the specimen was photographed in a location far from the known zone of sympatry between the two species, identification was based on geographic locality. We also personally examined specimens of *A. camura* and *A. bolivica* housed in the American Museum of Natural History and the Field Museum (USA), as well as in the Coleção Zoológica da Universidade Federal de Mato Grosso do Sul (Brazil). Imprecise records, such as state or country centroids, as well as dubious and duplicated catalog number records, were discarded from our database.

**FIGURE 1:**
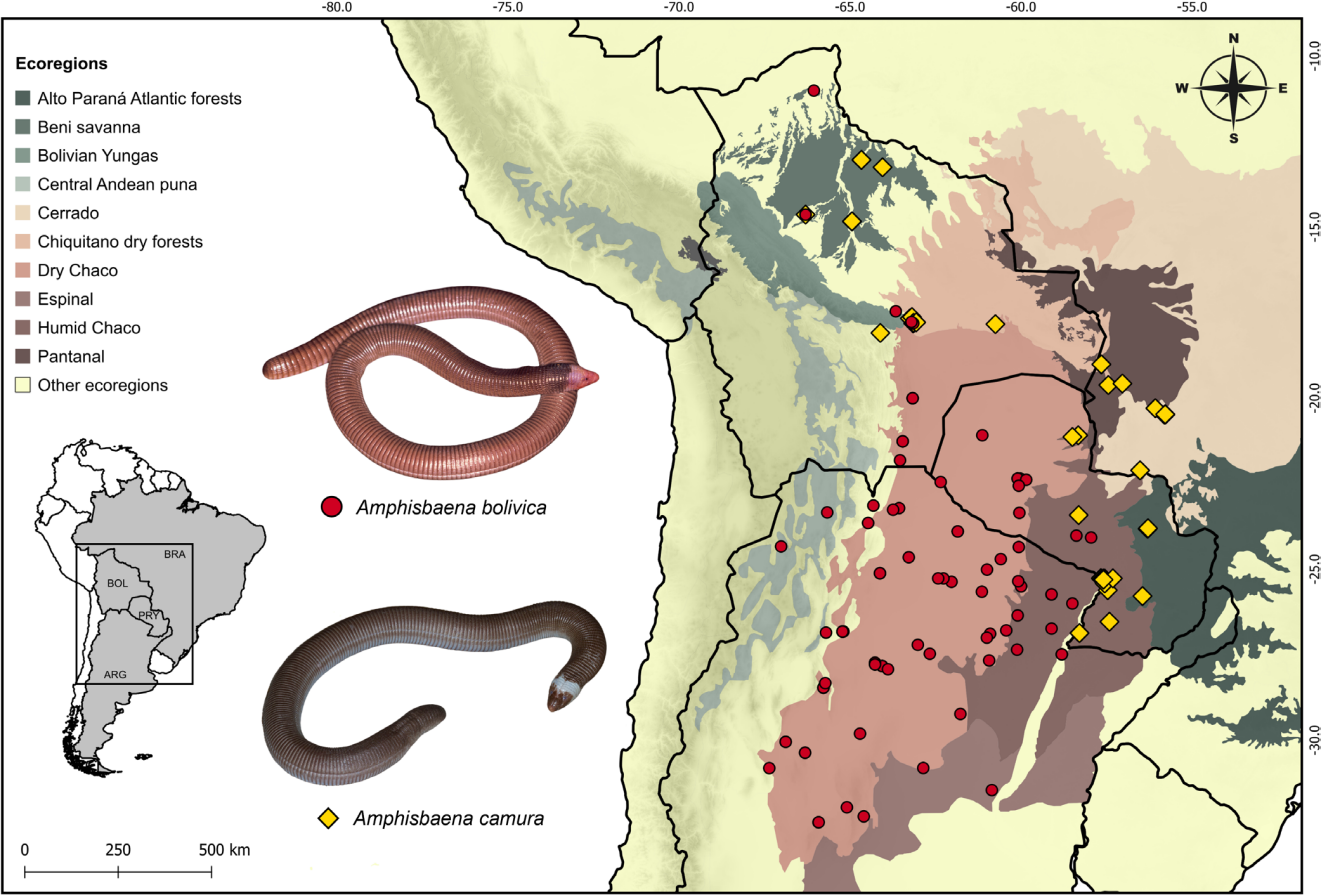
Map showing the known records and ecoregions of occurrence of *Amphisbaena bolivica* (red circles) and *Amphisbaena camura* (yellow diamonds). We standardized different symbols and sizes for the species points, aiming to facilitate the identification of sympatry areas. Individuals of *A. bolivica* (Photo by Paul Freed) and *A. camura* (Photo by Jean‐Paul Brouard—Fundación Para La Tierra) from Paraguay.

We obtained 125 records for *A. bolivica* (literature = 111, iNaturalist = 11, zoological collections = 3) and 126 for *A. camura* (literature = 98, iNaturalist = 11, zoological collections = 17) (Table S1). Using all available occurrence data, we produced a map illustrating the currently known distribution of each species using QGis v. 3.38 (QGIS Development Team 2024) (Figure 1). To reduce autocorrelation among occurrence data and potential for overfitting, we applied a spatial thinning procedure to exclude records falling within the same grid cells (~5 km), resulting in 66 records for *A. bolivica* and 33 records for *A. camura* . Computations were performed in R v. 4.4.2 (R Core Team 2024) using the package “spThin” v. 0.2 (Aiello‐Lammens et al. 2015).

### 2.2 Environmental Data

We used three sets of environmental factors: climate, edaphic, and slope. We downloaded 19 bioclimatic variables from the WorldClim database v. 2.1 at a resolution of 5 arc‐min (Fick and Hijmans 2017), averaged over the 1970–2000 period. The slope variable was based on the digital elevation model products of global 250 m GMTED2010 and near‐global 90 m SRTM4.1dev at a resolution of 2.5 arc‐min (Amatulli et al. 2018). We used edaphic variables of clay and sand content (g/kg), nitrogen (cg/kg), and soil organic carbon (dg/kg), which were downloaded from the SoilGrids database (Poggio et al. 2021) using weighted averages of the top three soil layers (0–30 cm in depth) at 250 m resolution. The slope and edaphic variables were resampled to a resolution of 5 arc‐min using the nearest neighbor interpolation in the “raster” R package v. 3.6 (Hijmans and Van Etten 2016).

### 2.3 Ecological Niche Modeling

We initially established the species accessible area to restrict predictions of habitat suitability to regions considered reachable by a species, defined by 400‐km buffers surrounding the occurrence data. While the 400‐km may be considered conservative, it also considers the potential limitations on data availability for fossorial species (Colli et al. 2016). Within the accessible area of each species, we generated a set of 10,000 random pseudo‐absence (PA) points. Next, we extracted environmental data across species records and performed a variable selection procedure to reduce multicollinearity among explanatory variables. For this purpose, we calculated the Variance Inflation Factor (VIF) among the explanatory variables, iteratively removing those with the highest VIF until all remaining variables showed VIF < 5. Computations were performed using the “usdm” R package v. 2.1‐7 (Naimi 2013). For *A. bolivica* we retained nine of 24 possible variables (Bio14, Bio18, Bio2, Bio3, Bio8, Clay, Sand, Soil organic carbon, and Nitrogen) and for *A. camura* we retained eight variables (Bio12, Bio18, Bio2, Bio3, Bio8, Clay, Sand, and Soil organic carbon) (Table S2).

We built an ENM using the Maximum Entropy algorithm (MaxEnt), one of the most commonly used and accurate algorithms for modeling the spatial distribution of living organisms (Phillips et al. 2006). MaxEnt was implemented using the “bigboss” optimization strategy in the “biomod2” R package v. 4.2‐6 (Thuiller et al. 2016). Variable importance was measured as each predictor's percentage contribution, reflecting its relative influence on model performance based on regularized gain. We used a five‐fold cross‐validation strategy to assess model performance, dividing the occurrence dataset into 80% species occurrence records as a training set and 20% as a test set, in addition to 10,000 randomly generated PA points. Individual model performance was evaluated using the true skill statistic (TSS) metric and the area under the ROC curve (Hanley and McNeil 1982; Elith et al. 2006), implemented in the “biomod2” R package. TSS values less than 0.4 are indicative of low predictive power, 0.4–0.8 of good predictive power, and 0.8 to 1 of excellent predictive power; values equal to or less than zero indicate performance no better than explained by chance (Zhang et al. 2015). The AUC values were classified as excellent at 0.90–1.00, good at 0.80–0.90, average at 0.70–0.80, poor at 0.60–0.70, or failure at < 0.60 (Zhang et al. 2015).

### 2.4 Niche Comparisons

Using ordination methods to assess niche overlap directly on environmental space is preferable to relying on habitat suitability values from ENMs, as projecting ENMs onto new areas can produce misleading patterns due to collinearity between relevant and irrelevant predictors (Broennimann et al. 2012). To obtain an orthogonal representation of environmental space, we conducted a principal component analysis (PCA) using all environmental predictors and then used the first two PCA axes to assess how each species occupies the environmental conditions available within its accessible area (Broennimann et al. 2012). This full background (all variables) is then compared to the areas effectively occupied by species across each of its ranges. Kernel density functions were used to produce smoothed densities of both occurrences and environmental availability (Broennimann et al. 2012).

We computed the niche overlap between *A. bolivica* and *A. camura* in the space defined by the environmental ordination using the Schoener's *D* statistic (Schoener 1968; Warren et al. 2008), which ranges from 0 (no niche overlap) to 1 (identical niches). Niche overlap analyses were conducted both in the full environmental space (combining all bioclimatic and edaphic variables) and separately for climatic and edaphic niches. This allowed independent testing of niche equivalence and similarity within each environmental dimension using PCA, Schoener's *D*, and null model tests.

We compared the observed value of *D* to two different null distributions to assess equivalency and similarity of ecological niches. To test niche equivalency, we combined records of both species and randomly split them into datasets of the same size as the originals, recalculating the niche overlap metric—*D* (Warren et al. 2010). We conducted 1000 iterations to build the expected distribution of *D* under the niche equivalence test, as this is typically sufficient to confidently reject the null hypothesis (Warren et al. 2008; Broennimann et al. 2012). The niche equivalence test is conservative and assesses if two species are identical in their niche space by using their exact locations, without considering the surrounding space.

To test niche similarity, we randomly shift the occurrence points of one species in environmental space and recomputed the Schoener's *D* metric across 1000 iterations. This generates an expected distribution of *D* under the null hypothesis that the observed niche overlap could occur by chance (Warren et al. 2010). A significant result would indicate not only that the two species occupy distinct environmental niches, but also that these differences are not merely due to geographic variation in the environment available. All analyses were performed using the R package “ecospat” v.4.1.2 (Di Cola et al. 2017).

## 3 Results

**FIGURE 2:**
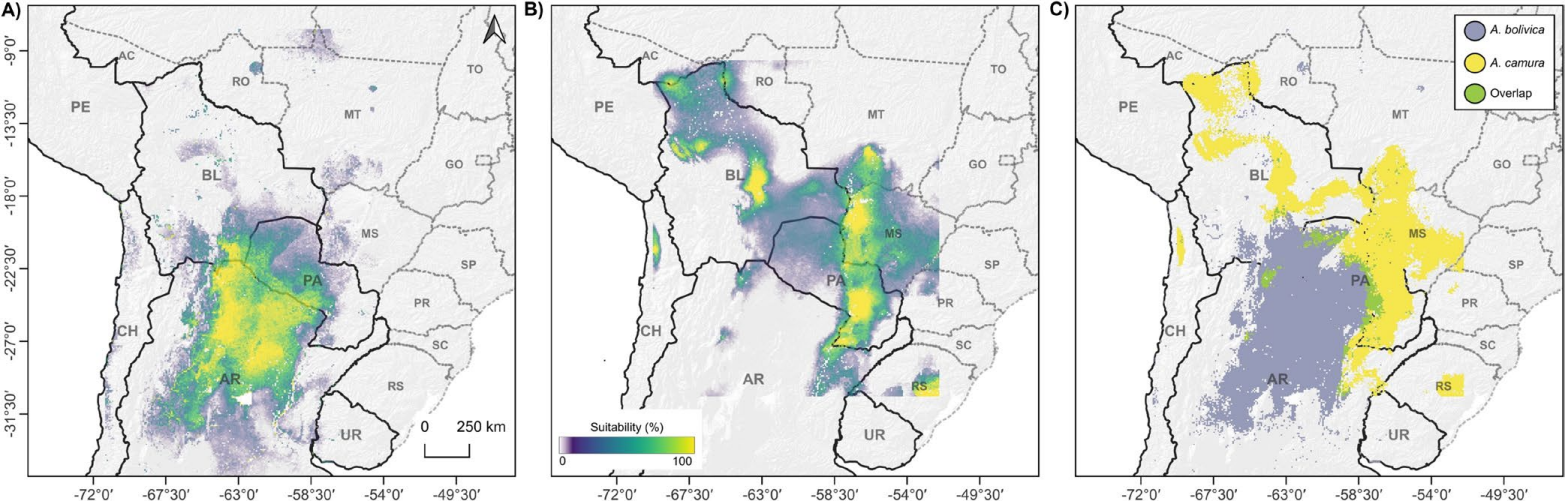
Ecological niche modeling for *Amphisbaena bolivica* (A), *Amphisbaena camura* (B), and the overlap distribution of the environmental niche of both species (C).

*Amphisbaena bolivica* primarily inhabits the Dry Chaco and Humid Chaco, but also occurs in other ecoregions, such as the Beni Savanna and the Chiquitano Dry Forests, whereas *A. camura* mostly exhibits a parapatric distribution pattern with relation to *A. bolivica* , with sympatry in parts of the Bolivian departments of Beni and Santa Cruz. *Amphisbaena camura* primarily occupies the Humid Chaco and Chiquitano Dry Forests, but also occurs in Beni Savanna, Cerrado, and Pantanal ecoregions (Figure 1). The performance of individual models yielded ROC values of 0.91, 0.90, and TSS values of 0.7, 0.71 for *A. bolivica* and *A. camura* , respectively (indicating a good predictive power). The projected habitat suitability for both species closely aligned with occurrence points. *Amphisbaena bolivica* model also shows suitable habitats in Brazil and Chile (Figure 2A). In contrast, there is a projection for *A. camura* potential occurrences in southern Mato Grosso, central Rio Grande do Sul (both in Brazil), and northern Chile (Figure 2B).

Based on the MaxEnt percentage contribution analysis, climatic variables were more important than soil and slope variables in determining the distribution of *A. bolivica* and *A. camura* (Table 1). Isothermality was the most important variable for the distribution of *A. bolivica* (67% contribution), followed by the mean temperature of the wettest quarter (43% contribution) and the precipitation of the driest month (26% contribution). The mean diurnal range was the most important variable for the distribution of *A. camura* (64% contribution), followed by the mean temperature of the wettest quarter (27% contribution) and isothermality (15% contribution). Other climate predictors contributed to the ENMs of *A. bolivica* and *A. camura* , though to a lesser extent. Edaphic variables less influenced their distributions, showing contribution < 10%.

**TABLE 1:** Summary of the selected variables for *Amphisbaena bolivica* and *Amphisbaena camura* and their respective relative importance.

Type	Abbreviation	Brief description	Percentage of contribution	
			*A. bolivica*	*A. camura*
Climatic	Bio2	Mean diurnal range (mean of monthly (max temp − min temp))	—	0.642
	Bio3	Isothermality (Bio2/Bio7) (×100)	0.676	0.155
	Bio8	Mean temperature of wettest quarter	0.437	0.272
	Bio12	Annual precipitation	—	0.131
	Bio14	Precipitation of driest month	0.265	—
	Bio18	Precipitation of warmest quarter	0.012	—
Edaphic	Clay	Clay content in g/kg	—	0.014
	Sand	Sand content in g/kg	0.109	0.049
	Carbon	Soil organic carbon in dg/kg	0.029	0.002
	Nitrogen	Nitrogen in cg/kg	0.050	—
Topographic	Slope	Average slope	0.052	—

**FIGURE 3:**
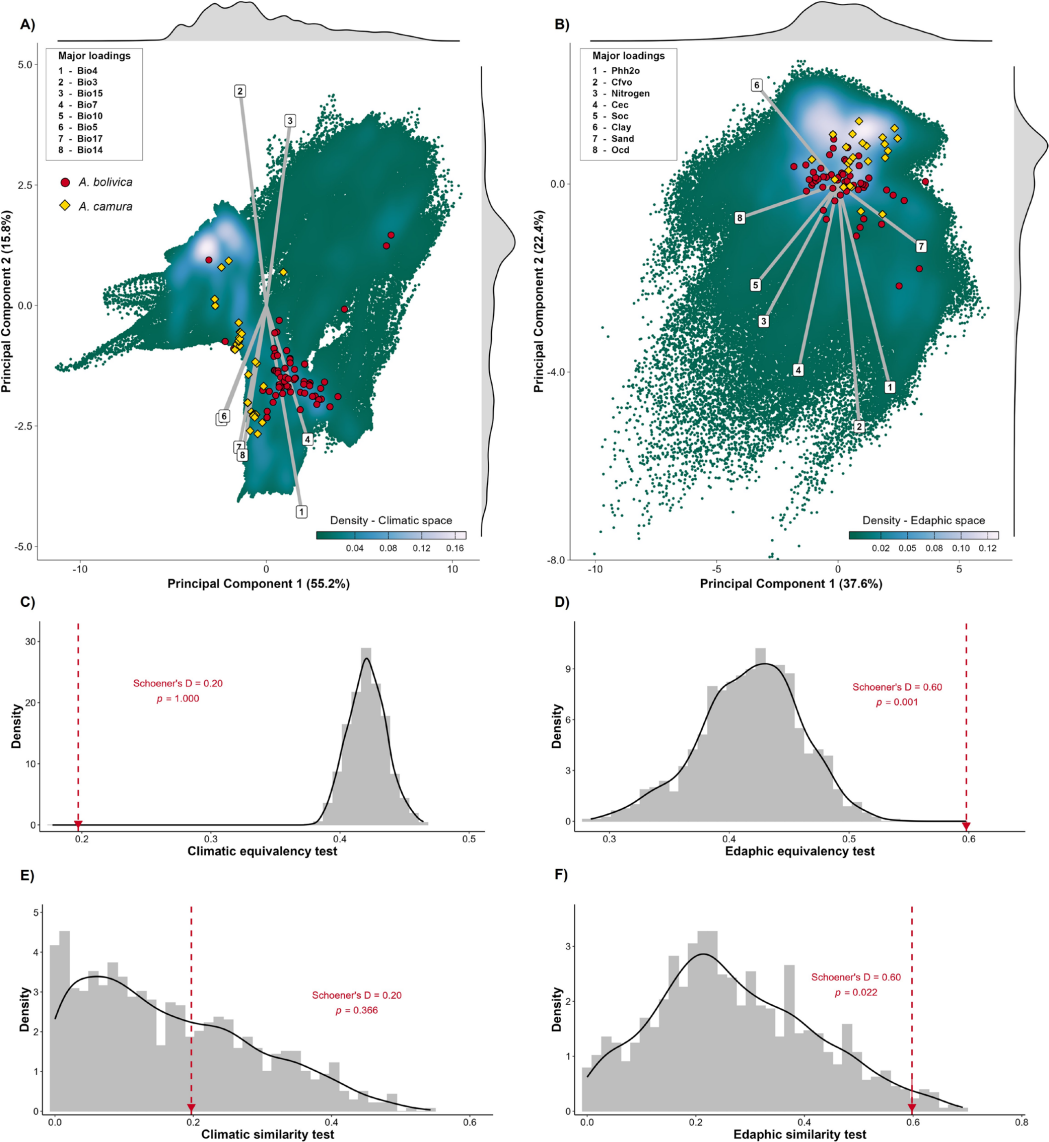
Comparative analysis of the ecological niche of *Amphisbaena bolivica* and *Amphisbaena camura* across different environmental spaces. Projection of species in the principal components of (A) climatic and (B) edaphic space. (C, D) Niche equivalency tests. (E, F) Niche similarity tests.

The observed Schoener's *D* value for overlap in the environmental space by these two *Amphisbaena* species was 0.20 (Figure 2C). This *D* value was lower than expected when compared to the null distribution generated for the test of either niche equivalence or similarity (*p* > 0.05) between *A. bolivica* and *A. camura* . Notably, the edaphic niche analyses between the species yielded higher overlap values than those observed in the climatic niche analyses. Additionally, the tests for niche equivalence and similarity in the edaphic space returned values exceeding the thresholds defined by the null models, indicating niche conservatism in the edaphic space (Figure 3, see also Figure S1).

## 4 Discussion

Fossoriality often reduces niche overlap through vertical niche partitioning but may also constrain diversification due to ecological specialization and reduced dispersal (Bars‐Closel et al. 2017; Cyriac and Kodandaramaiah 2018). For the worm lizards *Amphisbaena bolivica* and *A. camura* , we demonstrate that despite their close phylogenetic relationship, their environmental niches diverge climatically but converge edaphically. This segregation in the climate space is evidenced by the parapatric distribution, with sympatry occurring in regions of higher humidity and greater annual precipitation (Southwest Amazon moist forests, in northern Bolivia), but also in drier areas (Chiquitano dry forests, in southeastern Bolivia), ultimately pointing to a strong role of temperature in driving niche divergence, reinforcing the reliance of ectotherms on external heat sources to regulate body temperature and activity (Meiri et al. 2013; Luo et al. 2012; Buckley and Jetz 2010). Overall, our findings indicate limited opportunities for sympatric coexistence in these sister species of worm lizards, likely a result of their high conservatism of the edaphic space, in spite of their segregation in the climatic space.

Climatic variables, particularly temperature and precipitation, were the primary determinants of both species' geographic distributions, consistent with patterns observed in other fossorial squamates (Almeida et al. 2020; Oliveira et al. 2024; Machado et al. 2023). For *A. bolivica* , isothermality (Bio3) and mean temperature of the wettest quarter (Bio8) were the strongest predictors, aligning with its occurrence in the highly seasonal Dry Chaco (Figure 2A), where extreme annual temperature contrasts (summer: 49°C; winter: −7°C) coincide with low diurnal variation (Prohaska 1976). Such conditions likely reduce the energetic costs of burrowing by minimizing the need for vertical thermoregulatory movements (Şahin et al. 2021; Barros et al. 2021). In contrast, *A. camura* distribution was most influenced by mean diurnal range (Bio2) and wet‐season temperatures (Bio8), indicating adaptation to environments with pronounced day‐night temperature fluctuations, as seen in the Pantanal and Humid Chaco (Figure 2B). These climatic divergences suggest that *A. bolivica* is more tolerant of seasonal aridity, while *A. camura* may rely on wet‐season temperature for metabolic efficiency (O'Donnel and Ignizio 2012; Moreno‐Lara et al. 2023; Şahin et al. 2021; Muller et al. 2024).

Edaphic factors played a secondary role in niche differentiation. This finding aligns with our initial expectation that soil properties would show high overlap due to shared evolutionary constraints on burrowing adaptations (Vidal et al. 2008). The observed equivalency in the edaphic niche of *A. bolivica* and *A. camura* (Figure 3D) suggests that soil‐related factors represent a “filter” limiting dispersal or diversification in worm lizards. Moreover, similar patterns have been reported in other vertebrates, including fossorial taxa, where niche partitioning occurs along temporal, trophic, or climatic dimensions, while microhabitat conditions remain conserved (Székely et al. 2017; Di Pietro et al. 2020; Vacheva et al. 2025). A high overlap between species niches may arise under limited spatial variability of the available environmental conditions, meaning that niche similarity could arise solely by chance (Warren et al. 2010). That is not the case for *A. bolivica* and *A. camura* , which showed higher than random niche similarity (Figure 3F). Overall, our findings indicate that these worm lizards are adapted to a particular set of edaphic conditions, and likely face dispersal constraints along unsuitable soil gradients.

The lack of climatic niche overlap (Figure 3C), coupled with parapatric distributions, supports the recognition of *A. bolivica* and *A. camura* as distinct species. Their segregation in the climatic space aligns with the competitive exclusion principle (Hardin 1960), where niche partitioning facilitates coexistence (Şahin et al. 2021; Sudré et al. 2024). Notably, this challenges assumptions of phylogenetic niche conservatism (Wiens et al. 2010), as these closely related species exhibit divergent climatic preferences while retaining similar edaphic requirements. While these worm lizards strongly segregate along thermal‐related preferences, they tend to occupy somewhat similar hydric‐related conditions (Figure 3A), which helps explain their random patterns of climatic similarity (Figure 3E). This may reflect the lower risk of dehydration in subterranean microhabitats compared to surface environments, where evapotranspiration is higher; consequently, precipitation becomes a less critical factor for these fossorial species (Moore et al. 2018; Martín et al. 2024). This dichotomy in climate‐soil roles underscores the multifaceted nature of niche evolution in fossorial taxa, where certain ecological axes (e.g., climate) may diverge rapidly, while others (e.g., soil) remain conserved.

Our findings are tempered by limitations inherent to data availability, including sampling bias and the difficulty of detecting fossorial animals. We minimized these issues by incorporating expert‐verified records from the literature and citizen science platforms, along with examined museum specimens. Our assessment focused on the use of broad‐scale environmental conditions to characterize species niche, but we recognized that other unassessed finer‐scale variables likely mediate species coexistence, including vegetation‐related and micro‐soil conditions. While species coexistence can also result from temporal and trophic niche partitioning, lizards typically do not partition the trophic niche (Luiselli 2008; Sutherland 2011), including here worm lizards (Martín et al. 2013), with temporal activity patterns often linked to climate (Cohen et al. 2018; Jesus et al. 2023). Therefore, the strong role of climate in mediating worm lizard coexistence is likely robust across different niche dimensions.

The contrasting climatic niches of *A. bolivica* and *A. camura* highlight the role of environmental heterogeneity in driving divergence among fossorial reptiles. By partitioning climatic space while conserving edaphic requirements, these species exemplify how closely related fossorial reptiles can adapt to divergent climatic conditions to limit their competition in parapatric zones. Although fossoriality can facilitate species persistence under periods of climatic instability (Oliveira and Scheffers 2019), our findings also indicate that edaphic constraints reduce the ability of fossorial species to track suitable climates.

## Author Contributions

**Henrique J. Oliveira:** conceptualization (equal), data curation (equal), formal analysis (lead), investigation (lead), methodology (lead), writing – original draft (lead), writing – review and editing (lead). **Karoline Ceron:** conceptualization (equal), formal analysis (supporting), investigation (equal), methodology (equal), validation (equal), writing – review and editing (equal). **Mario R. Moura:** conceptualization (equal), methodology (equal), validation (equal), visualization (equal), writing – review and editing (equal). **Henrique C. Costa:** conceptualization (equal), data curation (equal), investigation (equal), methodology (supporting), project administration (lead), supervision (lead), validation (equal), writing – original draft (equal), writing – review and editing (equal).

## Conflicts of Interest

The authors declare no conflicts of interest.

## Supporting information


**Figure S1:** Second round (reciprocal test) of climatic and edaphic niche similarity for *Amphisbaena bolivica* and *Amphisbaena camura* .
**Table S1:** Occurrence records for *Amphisbaena bolivica* and *Amphisbaena camura* .
**Table S2:** Retained variables for *Amphisbaena bolivica* and *Amphisbaena camura* after thinning procedures and their Variance Inflation Factor (VIF) values.
**Appendix S1:** References from specialized literature used as data sources to compile the occurrence database of *Amphisbaena bolivica* and *Amphisbaena camura* . For details see Table S1.

## Data Availability

The raw data and R code needed to replicate the findings of this study are available at Zenodo Digital Repository: https://doi.org/10.5281/zenodo.15114608.
