# Supplementary material for: Climate, Not Soil, Drives the Distribution of Two Closely Related Worm Lizards
Source: Ecol Evol. 2025 Aug 15;15(8):e72008. doi: 10.1002/ece3.72008 (PMC12356706; doi:10.1002/ece3.72008)
Supplement: Supplementary file 1 — Figure S1: Second round (reciprocal test) of climatic and edaphic niche similarity for Amphisbaena bolivica and Amphisbaena camura . Table S1: Occurrence records for Amphisbaena bolivica and Amphisbaena camura . Table S2: Retained variables for Amphisbaena bolivica and Amphisbaena camura after thinning procedures and their Variance Inflation Factor (VIF) values. Appendix S1: References from specialized literature used as data sources to compile the occurrence database of Amphisbaena bolivica and Amphisbaena camura . For details see Table S1. [file ECE3-15-e72008-s001.zip › Supporting-Information_Oliveira-et-al_ECE-2025-03-00823.docx]

**SUPPORTING INFORMATION**

**Climate, not soil, drives the distribution of two closely related worm lizards**

Henrique J. Oliveira^*^, Karoline Ceron, Mario R. Moura and Henrique C. Costa

^*^Corresponding: henrique.bio22@gmail.com

**Appendix S1.** References from specialized literature used as data sources to compile the occurrence database of *Amphisbaena bolivica* and *Amphisbaena camura*. For details see Table S1.

Álvarez, B. B., García, J. A. R., Céspedez, J. A., Hernando, A. B., Zaracho, V. H., Calamante, C. C., & Aguirre, R. H. (2009). Herpetofauna, provinces of Chaco and Formosa, Chaco Oriental region, north-eastern Argentina. *Check List*, 5(1), 074-082. http://dx.doi.org/10.15560/5.1.74

Bucher, E. H. (2019). Amphibians and Reptiles. *The Mar Chiquita Salt Lake (Córdoba, Argentina) Ecology and Conservation of the Largest Salt Lake in South America*, 65-71.

Boulenger, G. A. (1894). XXXVIII List of reptiles and batrachians collected by Dr. J. Bohls near Asuncion, Paraguay. *Journal of Natural History*, *13*(76), 342-348. https://doi.org/10.1080/00222939408677709

Burt, C. E., & Burt, M. D. (1931). South American lizards in the collection of the American Museum of Natural History. *American Museum of Natural History*, 61, 227-395. https://doi.org/10.5479/si.00963801.2849

Cacciali, P., Scott, N. J., Aquino Ortíz, A. L., Fitzgerald, L. A., & Smith, P. (2016). The reptiles of Paraguay: literature, distribution, and an annotated taxonomic checklist. *Special Publication of the Museum of Southwestern Biology*, 11, 1-373.

Colli, G. R., Fenker, J., Tedeschi, L. G., Barreto-Lima, A. F., Mott, T., & Ribeiro, S. L. (2016). In the depths of obscurity: Knowledge gaps and extinction risk of Brazilian worm lizards (Squamata, Amphisbaenidae). *Biological Conservation*, 204, 51-62. https://doi.org/10.1016/j.biocon.2016.07.033

Cope, E. D. (1862). Catalogues of the reptiles obtained during the explorations of the Parana, Paraguay, Vermejo and Uraguay Rivers, By Capt. Thos. J. Page, USN; and of those procured by Lieut. N. Michler, US Top. Eng., Commander of the expedition conducting the survey of the Atrato River. *Proceedings of the Academy of Natural Sciences of Philadelphia*, 14, 346-594.

Dirksen, L., & De la Riva, I. (1999). The lizards and amphisbaenians of Bolivia (Reptilia, Squamata): checklist, localities, and bibliography. *Graellsia*, 55, 199-215. http://dx.doi.org/10.3989/graellsia.1999.v55.i0.329

Falcione, A. C., & Hernando, A. B. (2010). A new karyotypic formula for the genus *Amphisbaena* (Squamata; Amphisbaenidae). *Phyllomedusa*, 9(1), 75-80. http://dx.doi.org/10.11606/issn.2316-9079.v9i1p75-80

Gans, C. (1965). Notes on amphisbaenids. 17. Redescription and discussion of *Amphisbaena angustifrons* Cope and *A. camura* Cope, large amphisbaenids of southern South America (Amphisbaenia: Reptilia). *American Museum Novitates*, 2225, 1-32.

Gómez Alés, R., Galdeano, A. P., Acosta, J. C., & Blanco, G. M. (2019). *Amphisbaena bolivica* Mertens, 1929 (Squamata: Amphisbaenidae): Primer registro para la provincia de San Juan, Argentina. *Cuadernos de Herpetología*, 33(1), 39-40. http://dx.doi.org/10.31017/CdH.2019.(2018-025)

Hellmich, W. (1960). *Die Sauria des Gran Chaco und seiner Randgebiete* (Vol. 101). Verlag der Bayerischen Akademie der Wissenschaften.

Hicks, G., Vera Burró, A., Wang, J., Dickens, J., Davis, H.-P., Brouard, J.-P., Ríos, S. D., Santacruz, J. A., & Smith, P. (2022). New and significant distributional records of Paraguayan reptiles and amphibians from Alto Paraguay, Misiones, Ñeembucú, Presidente Hayes and San Pedro departments. *Historia Natural*, 12(3), 21-36.

Kacoliris, F. P., Berkunsky, I., & Williams, J. D. (2006). Herpetofauna of the argentinean impenetrable Great Chaco. *Phyllomedusa*, 5(2), 149-157. https://doi.org/10.11606/issn.2316-9079.v5i2p149-157

Lavilla, E. O., Cruz, F. B., & Scrocchi, G. J. (1995). Amphibiens et reptiles de la station biologique Los Colorados dans la province de Salta, Argentine. II. *Revue française d'aquariologie (Nancy)*, 22(3-4), 117-128.

Lavilla, E. O., Gonzáles, L., & Fernán-dez, I. (1996). Herpetología Amboroense. Informe sobre la herpetofauna del Parque Nacional Amboró y áreas aledañas. *Fundación amigos de la naturaleza/the nature conservancy, santa cruz de la sierra, Bolivia*.

Martínez Retta, L., Pardo, V. M., & Olivieri Bornand, S. E. (2023). Primer registro de *Amphisbaena bolivica* (Squamata: Amphisbaenidae) en San Luis, Argentina. *Cuadernos de Herpetología*, 37(1), 97-98. http://dx.doi.org/10.31017/CdH.2023.(2022-031)

Mertens, R. (1929). Herpetologische Mitteilungen. XXIII. Über einige Amphibien und Reptilien aus Süd-Bolivien. *Zoologischer Anzeiger*, 86, 57-62.

Montero, R. (1996). *Amphisbaena bolivica* Mertens 1929, nueva combinación (Squamata: Amphisbaenia). *Cuadernos de Herpetología*, 9, 75-84.

Montero, R., & Terol, G. (1999). Los Amphisbaenidae en Paraguay, listado geográfico. *Cuadernos de Herpetología*, 13, 89-95.

Peracca, M. G. (1897). *Rettili ed anfibi: viaggio del dott. Alfredo Borelli nel Chaco boliviano e nella Repubblica Argentina*. 274(12), 1-19.

Pesci, G. P., Sánchez, J. M., Muniz Leão, S., & Pelegrin, N. (2018). Reptiles y anfibios de una localidad del Chaco Húmedo en Formosa, Argentina. *Cuadernos de Herpetología*, 32(1), 47-54. http://dx.doi.org/10.31017/CdH.2018.(2017-05)

Ramallo, G., Bursey, C. R., & Goldberg, S. R. (2008). A new species of Cosmocercidae (Ascaridida) in the worm lizard, *Amphisbaena bolivica* (Squamata: Amphisbaenidae), from Argentina. *Journal of Parasitology*, 94(6), 1361-1363. https://doi.org/10.1645/GE-1415.1

Ribeiro, S., Sa, V., Santos-Jr, A. P., Graboski, R., Zaher, H., Guedes, A. G., ... & Vaz-Silva, W. (2019). A new species of the *Amphisbaena* (Squamata, Amphisbaenidae) from the Brazilian Cerrado with a key for the two-pored species. *Zootaxa*, 4550(3), 301-320. http://dx.doi.org/10.11646/zootaxa.4550.3.1

Roberto, I. J., Brito, L. B., & Avila, R. W. (2014). A new six-pored *Amphisbaena* (Squamata: Amphisbaenidae) from the coastal zone of northeast Brazil. *Zootaxa*, 3753(2), 167-176. http://dx.doi.org/10.11646/zootaxa.3753.2.6

Smith, P., Atkinson, K., Brouard, J. P., & Pheasey, H. (2016). Reserva Natural Laguna Blanca, departamento San Pedro: Paraguay's first Important Area for the Conservation of Amphibians and Reptiles?. *Russian Journal of Herpetology*, 23(1), 25-34. https://doi.org/10.30906/1026-2296-2016-23-1-25-34

Vanzolini, P. E. (1955). Contribuições ao conhecimento dos lagartos brasileiros da família Amphisbaenidae Gray, 1825. 5. Distribuição geográfica e biometria de *Amphisbaena alba*. *Arquivos do Museu Nacional*, 42(2), 683-705. https://doi.org/10.11606/0031-1049.1950.9p69-78

Vanzolini, P. E. (1968). Environmental temperature and number of body annuli in *Amphisbaena alba*: notes on a cline (Sauria, Amphisbaenidae). *Papéis Avulsos de Zoologia*, *21*(1-28), 231-241. http://dx.doi.org/10.11606/0031-1049.1968.21.p231-241

Werner, F. (1910). *Über neue oder seltene Reptilien des Naturhistorischen Museums in Hamburg: II. Eidechsen*. Gräfe & Sillem. 27(2), 1-48.

**Table S2.** Retained variables for *Amphisbaena bolivica* and *Amphisbaena camura* after thinning procedures and their Variance Inflation Factor (VIF) values.

| *A. bolivica* | |
| --- | --- |
| Retained variables | VIF |
| Slope | 3.13 |
| Bio14 | 2.72 |
| Bio18 | 2.62 |
| Bio2 | 3.14 |
| Bio3 | 1.91 |
| Bio8 | 3.41 |
| Clay | 2.57 |
| Sand | 2.96 |
| Carbon | 2.1 |
| Nitrogen | 1.92 |
|  |  |
| *A. camura* | |
| Retained variables | VIF |
| Bio12 | 1.88 |
| Bio18 | 1.87 |
| Bio2 | 1.52 |
| Bio3 | 1.54 |
| Bio8 | 1.37 |
| Clay | 3.08 |
| Sand | 2.46 |
| Carbon | 1.4 |

**Figure S1.** Second round of climatic and edaphic similarity tests for *Amphisbaena bolivica* and *Amphisbaena camura*.

| 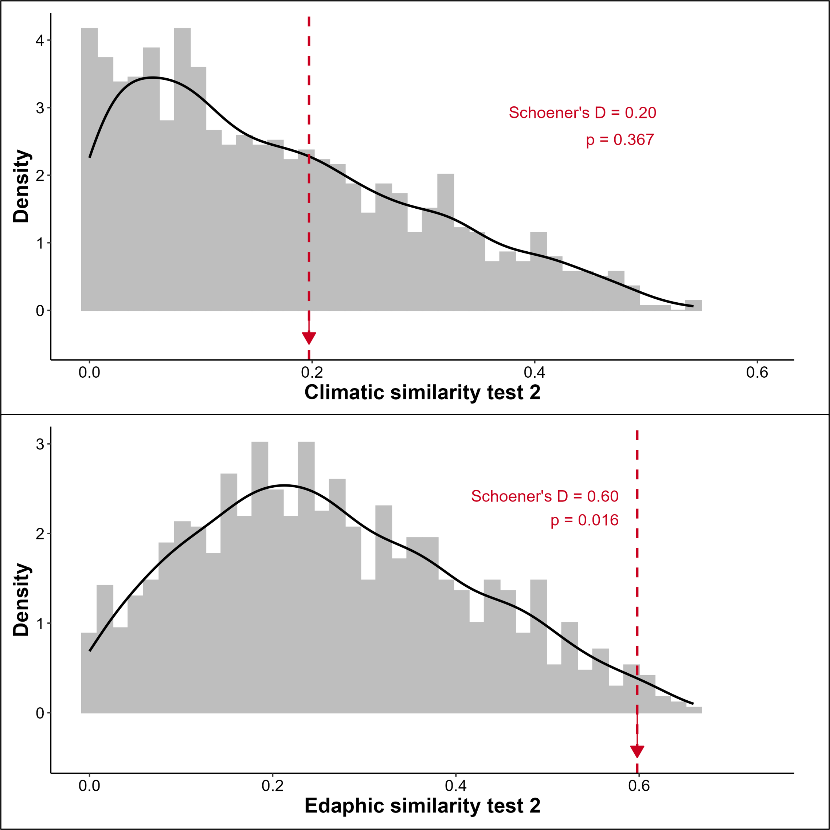 |
| --- |
